# Differentially expressed circulating LncRNAs and mRNA identified by microarray analysis in obese patients

**DOI:** 10.1038/srep35421

**Published:** 2016-10-21

**Authors:** Jia Sun, Yuting Ruan, Ming Wang, Rongping Chen, Na Yu, Lei Sun, Tiemin Liu, Hong Chen

**Affiliations:** 1 Department of Endocrinology, Zhujiang Hospital, Southern Medical University, Guangzhou, China; 2 Nephrology center of integrated traditional Chinese and Western Medicine, Zhujiang Hospital, Southern Medical University, Guangzhou, China; 3 The Second Clinical College of Southern Medical University, Guangzhou, China; 4 The Cardiovascular and Metabolic Disorders Program, Duke-NUS Graduate Medical School, Singapore; 5 The Third Affiliated Hospital, Harbin Medical University, Harbin, China; 6 Division of Hypothalamic Research, Department of Internal Medicine, UT Southwestern Medical Center, Dallas, Texas, USA

## Abstract

Circulating long non-coding RNAs (lncRNAs) serve as valuable biomarkers in a number of human diseases. However, lncRNA biomarkers have yet to be identified in obesity. We aim to characterize circulating lncRNA expression in obese and non-obese human subjects. First, we assessed the genome-wide circulating lncRNA expression profiles in blood from 3 obese and 3 non-obese human subjects. We found a significant decrease in circulating levels of three lncRNAs (lncRNA-p5549, lncRNA-p21015 and lncRNA-p19461) in obese human subjects only. Next, using RT-PCR we measured the expression levels of these three lncRNAs in 33 obese and 33 non-obese human subjects and found similar differences. Moreover, we found a negative correlation between circulating levels of these three lncRNAs and body mass index (BMI), waist circumference, waist to hip ratio and fasting insulin. There was also a significant negative correlation between expression of lncRNA-p19461 and homeostasis model assessment-estimated insulin resistance. Finally, we tested the circulating levels of these three lncRNAs in 8 obese human subjects after a 12-week diet-induced weight loss program. We found that only lncRNA-p19461 expression level significantly increased. In summary, circulating lncRNAs are deregulated in obesity. Weight loss–induced changes in this profile support this observation and suggest a potential mechanistic relevance.

Long noncoding RNAs (lncRNAs) are conventionally defined as a transcript longer than 200 nucleotides in length that lack protein-coding capability^1^. LncRNAs have been found in a stable form, protected from endogenous RNase activity, in tissues and body fluids including urine and blood^2^,^3^,^4^,^5^. The major role of lncRNAs is to regulate cell growth, proliferation, differentiation, and apoptosis^6^,^7^,^8^,^9^,^10^,^11^. Recent deep sequencing of human exosomes has shown that lncRNAs are localized within micro-vesicles, suggesting that lncRNAs may also play an important role in intercellular communication networks^12^. lncRNAs have also been implicated in the metabolic effects of insulin and the development of insulin resistance. Ellis *et al*. observed that insulin and insulin-like growth factor (IGF) 1 signaling triggered distinct changes in lncRNA CRNDE expression^13^.

lncRNAs serve as valuable biomarkers in a number of human diseases. For example, lncRNA transcript MVIH has been shown to be significantly up-regulated in hepatocellular carcinoma (HCC), suggesting that it might be an attractive biomarker for risk prognostication and personal therapy screening of HCC patients after hepatectomy^14^. Additionally, lncRNA transcript H19 is highly expressed in gastric cancer and is correlated with TNM cancer staging^15^,^16^. The expression of lncRNA transcript DD3 (PCA3) in urine also serves as a very sensitive and specific marker to detect prostate tumors, which may be used for clinical application^17^. Further evidence of the utility of lncRNAs in diagnosing/prognosing disease includes the dynamic change of the mitochondrial long noncoding RNA (LIPCAR) during myocardial infarction that predicted future death in patients with heart failure^18^. More recently, Zhao and Lin reported significant and specific regulation of 175 lncRNAs during adipogenesis using global transcriptome profiling of undifferentiated and mature adipocytes from the WAT and BAT lineages^19^. Finally, using Illumina microarrays, Divoux *et al*. identified a lncRNA (HOTAIR) that was expressed in gluteal adipose, but not in abdominal adipose tissue in 35 obese subjects, suggesting that lncRNAs may regulate key processes in adipocyte differentiation^20^. To the best of our knowledge, no data are available on changes in expression levels of circulating lncRNA in the obese state. With an unprecedented rise in worldwide obesity and its associated diseases, linking changes in levels of individual lncRNAs and obesity would be very valuable. It is well established that obesity is associated with lower quality of life, reduced life expectancy and increased risk of type 2 diabetes^21^, fatty liver disease^22^, cardiovascular disease^23^, and cancers^24^. Risk of obesity is likely determined by genetic makeup in close relationship with behavioral and environmental factors^25^,^26^,^27^. However, the exact pathogenesis of this disease remains elusive and calls for a broader view of the complex systems of energy balance regulation. Identification of accurate and reproducible noninvasive metabolic biomarkers of obesity may advance prevention, diagnosis and treatment options for the disease. Increasing evidence indicates that lncRNAs may provide insight into regulating gene expression related to obesity^28^. In the current study, we investigate the expression profile of lncRNAs and related mRNAs in obese patients, with the broader goal of identifying obesity-affecting genes and understanding the pathogenesis of obesity.

## Results

### Comprehensive circulating lncRNAs profiling in the blood samples from obese and non-obese participants

**Figure 1:**
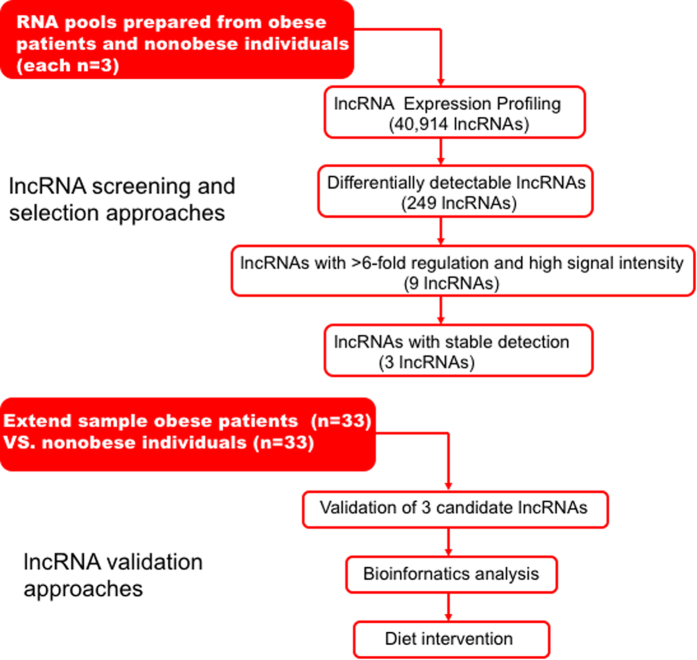
Flowchart of long noncoding RNAs (lncRNAs) screening and validation in obese and non-obese participants.

**Figure 2:**
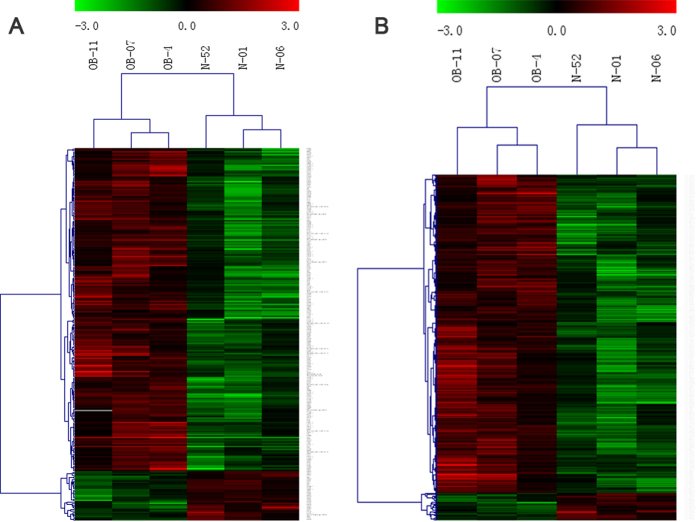
Expression profiles of differentially expressed lncRNAs and mRNAs in obese and non-obese participants. Differentially expressed lncRNAs and mRNAs between obese and non-obese participants were subjected to hierarchical clustering. The color scale on the top illustrates the relative expression level of lncRNAs across all samples. Red color indicates high relative expression and green color indicates low relative expression. (**A**) lncRNA; (**B**) mRNA.

**Figure 3:**
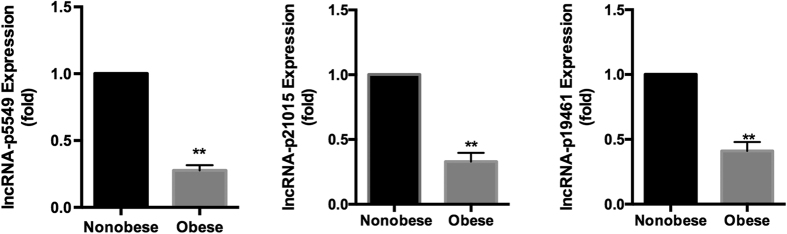
Expression level of circulating lncRNA-p5549, lncRNA-p21015 and lncRNA-p19461 in obese and non-obese participants. Expression of lncRNA-p5549, lncRNA-p21015 and lncRNA-p19461 was detected by qPCR and normalized by U6 expression. (**P < 0.01)

We designed a pilot study to identify the circulating lncRNA signature in obese patients (Fig. 1). We tested whether any significant changes in lncRNA expression was observed in obese and non-obese participants’ blood samples. lncRNA arrays were performed using RNA derived from whole blood sample of participants. Table 1 summarizes anthropometric and metabolic characteristics of study participants. The lncRNA expression patterns between 3 obese and 3 non-obese blood samples were found to be significantly different. Using a 2-fold expression difference as a cutoff, a total of 249 lncRNA transcripts were specifically dysregulated (213 lncRNA transcripts upregulated and 36 lncRNA transcripts downregulated; each *p* < 0.05) in obese participants, compared with non-obese participants. Additionally, a total of 393 mRNAs were specifically dysregulated among 34,235 mRNA including 362 mRNA upregulated and 31 mRNA downregulated (Fig. 2, Online Supplemental Materials 1). Filtering of all dysregulated transcripts for high signal intensity and ≥6-fold deregulation yielded 9 lncRNA candidates. Only 3 (lncRNA-p5549, lncRNA-p21015 and lncRNA-p19461) of these 9 lncRNA candidates could be consistently dysregulated in a larger cohort of blood samples (33 obese participants and 33 healthy non-obese control participants). The expression levels of circulating lncRNA-p5549, lncRNA-p21015 and lncRNA-p19461 in obese participants were significantly decreased compared with non-obese participants (Fig. 3, *p* = 0.004, *p* = 0.002 and *p* = 0.002, respectively). They were also negatively associated with BMI (*p* < 0.01 for all), waist circumference and waist to hip ratio (Table 2). Moreover, the expression level of circulating lncRNA-p19461 was negatively associated with HOMA-IR. After adjusting the important cofactors of HOMA-IR and found that the expression levels of circulating lncRNA-p19461 in obese participants remained significantly decreased compared with non-obese participants (*p* = 0.014). Taken together, these results suggest a link between obesity and the level of circulating lncRNAs.

### Tissue expression pattern of the circulating lncRNA-p5549, lncRNA-p21015 and lncRNA-p19461 in blood components and adipose tissue

**Figure 4:**
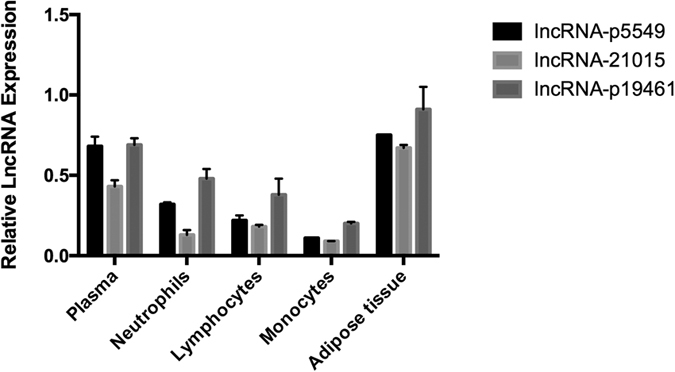
Different expression of three lncRNAs in 4 components sorted from peripheral blood and adipose tissue of obese patients.

Since we extracted RNA from whole blood sample, we needed to explore further the origin of total RNA. As shown in Fig. 4, expression levels of these three lncRNAs in adipose tissue far outweighed that in peripheral blood. Additionally, these lncRNAs mainly existed in plasma rather than haemocytes.

### Effect of weight loss on the expression levels of circulating lncRNA-p5549, lncRNA-p21015 and lncRNA-p19461

**Figure 5:**
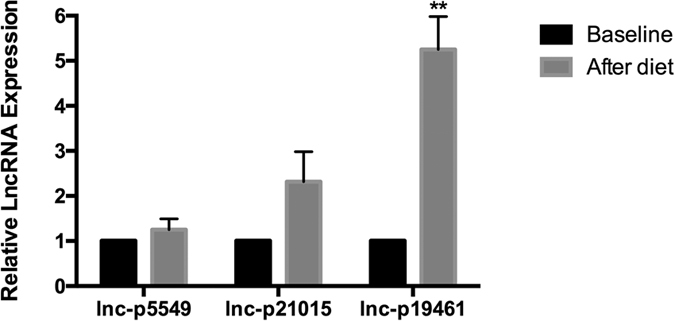
Baseline and diet-induced weight loss levels of lncRNA-p5549 (**A**) lncRNA-p21015 (**B**) and lncRNA-p19461 (**C**) in obese participants. **P <0.01. Date are shown as mean (SD).

Dietary intervention of very low carbohydrate diet (VLCD) is a feasible alternative recommendation for weight loss^29^. Emerging evidence has consistently shown that a VLCD can protect against the development of obesity, but the underlying mechanisms are not fully understood^30^. Nine of the 33 obese participants agreed to take part in VLCD and were placed on a 12-week diet-induced weight loss program. One participant dropped out because of nausea, leaving 8 remaining obese participants (4 males and 4 females) in the final analysis. After 12 weeks, these 8 obese participants lost on average approximately 8% of their initial body weight (Table 3). We observed sex differences of VLCD weight loss; the effects of weight loss were more obvious in females than in males (mean difference of BMI, −3.85 vs. −1.68, p = 0.013). We tested the expression levels of circulating lncRNA-p5549, lncRNA-p21015 and lncRNA-p19461 in plasma samples at 12 weeks post-VLCD. The expression levels of circulating lncRNA-p5549 and lncRNA-p21015 were not significantly different compared to pre-VLCD levels. In contrast, the expression level of circulating lncRNA-p19461 was significantly upregulated (Fig. 5).

### Gene Enrichment and Pathway Analysis of lncRNA-Coexpressed mRNA

**Figure 6:**
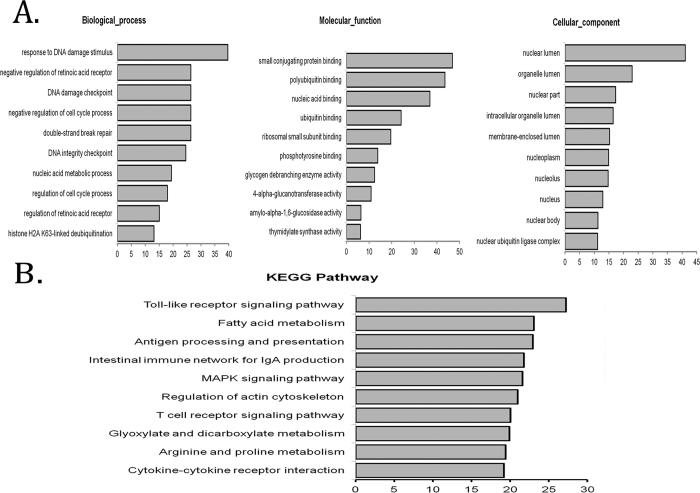
Gene enrichment and pathway analysis of lncRNAs–coexpressed mRNAs. (**A**) The gene ontology enrichment analysis provided a controlled vocabulary to describe the differentially expressed lncRNAs–coexpressed mRNAs. The ontology covered three domains: biological process, cellular component, and molecular function (P < 0.05 is recommended). (**B**) KEGG analysis suggested that lncRNAs–coexpressed mRNAs were mainly targeted to the Toll-like receptor signaling pathway.

Gene enrichment analysis was performed to determine the gene and gene product enrichment in biological processes, cellular components, and molecular functions^31^. As shown in Fig. 6A, we found that the highest enriched GOs targeted by lncRNAs–coexpressed mRNAs were responses to DNA damage (ontology: biological process), stimulus small conjugating protein binding (ontology: molecular function), and nuclear lumen (ontology: cellular component). Kyoto Encyclopedia of Genes and Genomes (KEGG) pathway analysis indicated that the lncRNAs–coexpressed mRNAs were involved in the regulation of Toll-like receptor signaling pathway, fatty acid metabolism, antigen processing and presentation, intestinal immune network for IgA production, and MAPK signaling pathway (Fig. 6B).

### Construction of lncRNA/mRNA Coexpression Network

**Figure 7:**
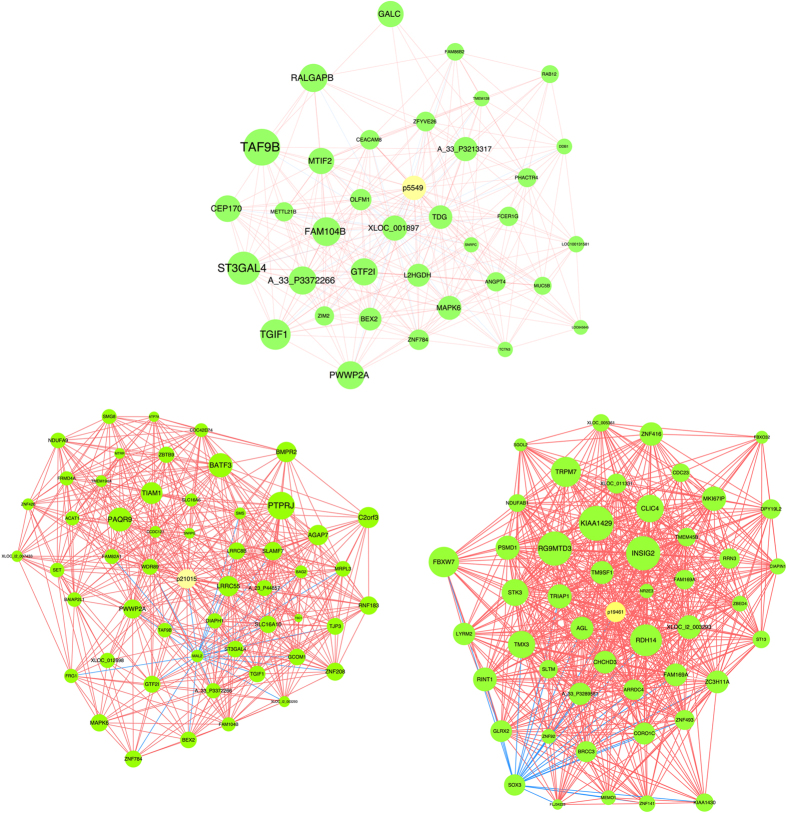
Co-expression networks of obesity-associated genes and co-regulated lncRNAs. 145 mRNAs interacted with three lncRNAs. Genes colored in yellow are lncRNAs, genes colored in green are mRNA. Red line represents positive correlation. Blue line represents negative correlation. Node size represents the node degrees.

The microarray data also provided information on mRNA expression between obese and non-obese participants. We constructed the lncRNA/mRNA coexpression network and investigated the potential interaction between mRNAs and lncRNAs. One hundred forty-five mRNAs interacted with these three lncRNAs in 10 meaningful pathways. We drew the regulatory network of lncRNA-p5549, lncRNA-p21015 and lncRNA-p19461 using the cytoscape program (Fig. 7, Online Supplemental Materials 2). The network indicated that each lncRNA correlated with a large number of target mRNAs suggesting that the inter-regulation of lncRNAs and mRNAs occurred in the obese state only.

## Discussion

Knowledge of lncRNA species and biology is emerging rapidly, however bioinformatic strategies for identifying their functionality is still deficient^32^. There is limited knowledge of lncRNA regulation in human peripheral blood or their role in human disease. In this study, we identified 249 lncRNAs and 392 mRNAs abnormally expressed in blood samples of obese participants compared with non-obese participants (fold change ≥2.0, *p* < 0.05). We demonstrated that lncRNA-p5549, lncRNA-p21015 and lncRNA-p19461 were downregulated in obese patients compared with controls using qRT-PCR. In addition, there was a significant correlation between circulating levels of these three lncRNAs and BMI, waist circumference and waist to hip ratio. However, there was no observed association between these three lncRNAs and obesity-associated inflammation markers (interleukin-6 and C-reactive protein). We further detected the possible sources of the circulating lncRNAs in blood components (plasma, neutrophils, lymphocytes and monocytes) and adipose tissue. The expression level of these three lncRNAs in adipose tissue far outweighed that in peripheral blood. Additionally, these lncRNAs mainly existed in plasma instead of haemocytes. We speculate that these circulating lncRNAs may derive partly from adipose tissue, a target organ in the development of obesity. However, we found no correlation between the expressions of lncRNAs in adipose tissue and BMI, suggesting that lncRNAs might be tissue-specific and dynamically regulated in obese patients. Finally, we studied the effects of a 12-week dietary intervention on serum metabolic profiles and circulating lncRNAs expression. We found that the diet intervention resulted in weight loss and significantly decreased fasting insulin and decreased levels of HOMA-IR. Interestingly, the circulating level of lncRNA-p19461 was negatively associated with insulin resistance and upregulated by diet-induced weight loss. Therefore, our findings suggest that a change in body weight regulates the expression levels of circulating lncRNAs such as lncRNA-p19461. Future studies with larger sample sizes may confirm the role of other two candidate lncRNAs after diet-induced weight loss.

To obtain novel insights into the function of lncRNAs, we used gene ontology enrichment analysis to construct a co-expression network by combining differentially expressed lncRNAs with differentially expressed mRNAs. lncRNA-mRNA interactions were thoroughly surveyed and identified based on their locational distributions and sequence correlations. The two members of each mRNA-lncRNA pair can overlap completely or partially (on the same strand). Some of them can also form duplexes through reverse complementary binding (from the sense/antisense strands), which may facilitate interactions between different RNA molecules. The mRNA and lncRNA from the same strands sometimes show opposite deregulation trends. Abnormal regulation in the lncRNA-mRNA network may be pivotal to the development of obesity. The different levels of final enrichment may be attributable to the regulatory mechanisms. In our study, we found 145 mRNAs interacted with these three lncRNAs in 10 meaningful pathways. The most enriched network was ‘Toll-like receptor signaling pathway’, which is known as a pro-inflammatory pathway. Previous studies have demonstrated that activation of the pro-inflammatory pathway is linked to obesity and insulin resistance^33^,^34^. Fat acid metabolism was the second richest pathway observed, suggesting that lncRNAs may influence the risk of obesity by regulation of fat acid metabolism. This outcome suggests that the inter-regulation of lncRNAs and mRNAs may be involved in the development or progression of obesity, warranting further study to confirm the relationship and underlying regulatory mechanism of these coding and noncoding genes.

Additionally, according to the analysis of the gene pairs formed by lncRNAs and their neighboring genes, we found many significant correlations between lncRNAs and multiple protein-coding genes. Most evidence suggests that the expression of lncRNAs can regulate and have high correlations with expression of neighboring mRNAs^35^,^36^. Based on this, we searched coding genes 10 k/100 k upstream and downstream of lncRNA as the cis target genes and predicted the function of lncRNA. Consequently, we found that many lncRNAs might exert their function through predicted mRNA. For example, we found that lncRNA-p5549 was associated with thymine-DNA glycosylase (TDG), which is involved in DNA repair^37^,^38^, DNA demethylation^39^ and transcriptional regulation^40^. TDG can bind to DNA methylation enzymes to maintain normal methylation patterns, which might represent an important homeostatic mechanism of metabolism by epigenetic modifications. We also observed a coexistence phenomenon of p19461 and coiled-coil helix coiled-coil helix domain-containing protein 3 (CHCHD3). CHCHD3, an inner mitochondrial membrane protein, is essential for crista integrity, mitochondrial function and cellular energy metabolism^41^. Mitochondria are ancient organelles that are thought as critical elements of energy metabolism^42^. The impaired mitochondrial function damages the intracellular energy metabolism including physiological fat oxidation and promotes the accumulation of adipose tissue, increasing the risk of obesity. We speculate that p19461 may protect mitochondrial function through regulation of CHCHD3, accelerating fat oxidation and reducing the incidence rate of obesity. However, obese patients characterized by a high-fat high-glucose diet might result in downregulating of these lncRNAs, then reducing the energy consumption in the obese state. A genome-wide association study also identified CHCHD3 as promising gene for back fat in pigs^43^. Whether a similar association is found in humans remains to be investigated.

Our study has several limitations. The sample size for microarray analysis and verification was small and a larger sample cohort is needed to verify the identifications. In clinical practice, 5–10 ml is the typical volume drawn for a single blood draw. Unfortunately, this volume produces a low quantity of circulating mRNA in the blood sample. Similarly, the amount of tissue that could be feasibly obtained and the complexity of the tissue sample itself limited the quantity of tissue analyzed. Another possible limitation was the storage and transportation of samples which may have resulted in RNA degradation. Thus, the present study should be considered as hypothesis generating; further genetic and experimental investigations involving target genes and the onset of obesity is planned.

## Materials and Methods

### Study Subjects

Figure 1 illustrates the strategy of lncRNA screening and validation. The WHO recommends using BMI cut points to define obese and overweight adults. In China, BMIs of 24 kg/m^2^ and 28 kg/m^2^ are considered to be the cut points to classify normal weight, overweight and obesity^44^. We excluded individuals with microvascular diseases, hypertension, coronary disease, systemic inflammatory diseases, acute respiratory infection or cancers. To profile circulating lncRNAs, we recruited and drew blood samples from 3 obese patients (BMI ≥ 28 kg/m^2^) and 3 non-obese individuals (BMI < 24 kg/m^2^) from the Endocrinology Department, Zhujiang Hospital of Southern Medical University (Guangzhou, China).

We identified dysregulated lncRNAs in obese patients for further analysis and validation. The association between the selected lncRNAs and obesity characteristics and prognosis was then analyzed in the circulation of an expanded cohort of obese patients (n = 33) and nonobese controls (n = 33). To explore further the origin of lncRNAs in whole blood of obese patients, we recruited 10 obese patients about to undergo surgery in the Department of General Surgery, Zhujiang Hospital. We selected these patients from a consecutive series of patients with obesity. The Research Ethics Committee of Zhujiang Hospital of Southern Medical University (2014-NFMK-005) approved the study protocol. We obtained written informed consent from all participants for the use of their blood specimens. The entire trial was conducted in accordance with the guidelines of the Research Committee of Zhujiang Hospital of Southern Medical University (Guangzhou, China) and the blood samples and tissue specimens were handled according to the guidelines of the Helsinki Declaration.

### Blood and Tissue Collection

For lncRNA quantification, we collected a fasting venous blood sample (10 ml) from each participant at least 12 h following their most recent meal. Neutrophils were isolated by using improved Ficoll Solution (TBD). We isolated human peripheral blood mononuclear cells (PBMCs) using Lymphoprep (Axis-Shield, Norway). Monocytes were then enriched through positive immunomagnetic selection by using anti-CD14 MicroBeads (Miltenyi, Germany). Lymphocytes were isolated from PBMCs by fluorescence-activated cell sorting (Aria II Cell Sorter; BD Biosciences) according to manufacturer’s instructions. All fluorescence-activated cell sorting data analysis was performed using FlowJo software (Tree Star). All leukocytes were isolated <12 h after collection. Lipid and glucose measurements were automated using Siemens Advia 2400 Clinical Chemistry System (Siemens, Erlangen, Germany). Human insulin levels were measured using a human INS ELISA Kit (R&D Systems, Minneapolis, MN, USA). Immediately after surgery, we collected adipose tissues, washed in cold PBS, and stored in RNAlater RNA Stabilization Reagent (Qiagen) at −20 °C for use.

### RNA Extraction

Total RNAs were extraction from the fasting whole blood samples using TRIpure LS Reagent (Bioteke, Beijing, China) in accordance with the manufacture’s protocol, and stored at −80 °C before use and were thawed on ice before use. Total cellular RNA was isolated from the fresh adipose tissues using an RNeasy Mini Kit (Qiagen, Hilden, Germany) according to the manufacturer’s protocol. The RNA integrity of each sample was assessed using standard denaturing agarose gel electrophoresis.

### lncRNAs Microarray Profiling

We performed microarray profiling using Human LncRNA Microarray V4.0 (CapitalBio Corp, Beijing, China), including 34,235 mRNAs and 40,914 lncRNAs. Briefly, we labeled 10 μg of total RNAs using the Superscript Plus Direct cDNA labeling system (Invitrogen) followed by hybridization to the chip. We then scanned microarrays using an Agilent microarray scanner piloted by GenePix Pro 6.0 software (Axon). We imported scanned images (TIFF format) into Agilent Feature Extraction software for grid alignment and expression data analysis. Expression data were normalized by a quartile normalization and the Robust Multichip Average (RMA) algorithm that was included in the Agilent software. After normalization, we generated probe-level files and mRNA-level files. All gene-level files were imported into Agilent GeneSpring GX software (version 11.5.1) for further analysis. We identified differentially expressed lncRNAs and mRNAs through fold change filtering. The microarray data were selected by threshold values of >2 and <−2 fold change under FDR protection (P < 0.05).

### Validation of lncRNA Gene Expression in Blood by Real-time Quantitative PCR (RT-PCR)

We performed RT-PCR quantification of lncRNA expression using TaqMan lncRNA assays (Applied Biosystems Inc., Foster City, CA) according to the manufacturer’s protocol. We used the ABI Prism 7500 sequence detection system (Applied Biosystems, Foster City, CA) to perform RT-PCR. Briefly, reactions were performed in a mixture (20 μL) containing 5μL cDNA template, 10 μL 2 × SYBR-Green PCR Mix (TaKaRa), and 0.5 μL each of sense and antisense primers. Additional File 1 shows the sequences of primers used for RT-PCR. We performed RT-PCR in duplicate for each sample, and estimated the specificity of PCR product through the dissociation curve. U6 small nuclear RNA was the internal control. For quantitative results, expression of each lncRNA was represented as a fold change using the 2^−ΔΔCt^ method and then analyzed for statistical significance.

### Study of the Effects of Weight Loss

We performed a longitudinal validation study to investigate the expression levels of candidate lncRNAs before and after diet-induced weight loss in 9 obese patients (4 men and 5 women). We randomly selected this subpopulation from the cohort of 33 obese patients recruited at the Endocrinology Department of Zhujiang Hospital of Southern Medical University (Guangzhou, China). We gave selected obese patients individual instructions to follow the very low carbohydrate diet for 12 weeks. Energy intake was restricted to less than 1200 kcal/day (5021 kJ/d) (carbohydrate intake <20 g/d). We substituted carbohydrate-rich foods, such as white rice, steamed bread and tubers with fish, poultry and plant oil. We performed genomic measurements in plasma at baseline and 12 weeks after intervention start. We conducted the study protocol in accordance with the Declaration of Helsinki. The Ethics Committee and the Committee for Clinical Investigation of Zhujiang Hospital of Southern Medical University (2014-NFMK-005) approved the protocol. We obtained written informed consent from all patients after the purpose of the study was explained to them.

### Bioinformatics Analysis

To determine the roles that differentially expressed mRNAs played in biological pathways or GO terms, we applied pathway analysis and GO analysis. Differentially regulated mRNAs were uploaded into the Database for Annotation, Visualization and Integrated Discovery (DAVID, http://david.abcc.ncifcrf.gov/) for annotation and functional analysis, including gene set enrichment analysis and mapping gene sets to the KEGG pathway^45^. We also used the KEGG (Kyoto Encyclopedia of Genes and Genomes) database (http://www.genome.ad.jp/kegg) and BioCarta (http://www.biocarta.com) to analyze the potential functions of these target genes in the pathways^46^,^47^.

### lncRNA/mRNA Coexpression Network

To associate the lncRNAs with direct regulated expression of target mRNAs, we superimposed lncRNA target predictions onto the lncRNA-mRNA correlation network. We defined the resulting network as an lncRNA-mRNA regulatory network. The algorithm was quoted from a previously described report^48^. For each pair of gene analysis, the Pearson correlation was calculated and we chose those pairs with significant correlations (0.90 or greater) to construct the network. We drew the coexpression networks using Cytoscape. In this representation, each gene corresponded to a node and the connection of two genes was represented by an edge, indicating a strong correlation (i.e. either positive or negative).

### Statistical Analysis

Data were analyzed using SPSS version 22.0 software. Continuous variables were presented as means ± SD or median (interquartile range). More than 2-fold changes of individual lncRNAs were calculated for each subject by dividing the standardized expression levels of the lncRNAs. Differences in lncRNA levels between obese groups and control groups were analyzed using unpaired Student *t* test. *p* values less than.05 were considered statistically significant. To analyze interactions between potentially confounding effects of lifestyle features and other variables related to obesity, we additionally performed correlation analyses and logistic regression analyses for matched data that included expression levels of lncRNAs and the following variables: body mass index, waist circumference, waist-to-hip ratio, fasting glucose, fasting insulin, TC, TG, HDL cholesterol, LDL cholesterol, IL-6 and high-sensitivity C-reactive protein.

## Additional Information

**How to cite this article**: Sun, J. *et al*. Differentially expressed circulating LncRNAs and mRNA identified by microarray analysis in obese patients. *Sci. Rep.* **6**, 35421; doi: 10.1038/srep35421 (2016).

## Supplementary Material

Supplementary Dataset 1

Supplementary Dataset 2

## Figures and Tables

**Table 1:** Clinical characteristics of included individuals in cross-sectional studies.

	Nonobese (n = 33)	Obese (n = 33)	*p*
Age (years)	41.00 ± 12.71	35.77 ± 11.69	0.206
Sex (male/female)	17/16	17/16	1.000
BMI (kg/m^2^)	21.75 ± 1.74	32.66 ± 4.45	0.000
Waist circumference (cm)	76.22 ± 6.07	112.58 ± 11.14	0.000
Waist-hip Ratio	0.82 ± 0.05	0.96 ± 0.05	0.000
Fasting glucose (mmo/L)	4.83 ± 0.57	5.08 ± 0.54	0.045
Fasting insulin (μU/mL)	6.47 ± 3.24	16.85 ± 9.35	0.002
Fasting C-peptide (ng/mL)	1.58 ± 0.40	3.52 ± 1.45	0.001
HOMA-β (%)	136.52 ± 153.12	197.32 ± 113.96	0.260
HOMA-IR	1.42 ± 0.84	4.73 ± 2.45	0.001
Total cholesterol (mg/dL)	4.70 ± 0.78	4.87 ± 1.85	0.767
Fasting triglycerides (mg/dL)	1.43 ± 1.00	2.32 ± 1.09	0.040
HDL cholesterol (mg/dL)	1.39 ± 0.38	1.11 ± 0.22	0.031
LDL cholesterol (mg/dL)	3.04 ± 0.74	3.58 ± 1.17	0.170
IL-6 (Pg/ml)	4.52 ± 3.86	5.49 ± 4.07	0.636
CRP (mg/dl)	4.28 ± 8.49	6.46 ± 8.67	0.557
Fatty liver (%)	16.4%	78.9%	0.006

**Table 2:** Correlation between lncRNAs concentrations and studied variables in the cross-sectional study Data are R (*p*).

	BMI	Waist circumference	Waist to hip ratio	Fasting glucose	Fasting Insulin	HOMA-IR	HOMA-β	IL-6	CRP
lncRNA-p5549	−0.528 (0.008)	−0.467 (0.019)	−0.439 (0.029)	−0.214 (NS)	−0.423 (0.041)	−0.386 (NS)	−0.269 (NS)	−0.277 (NS)	−0.045 (NS)
lncRNA-p21015	−0.467 (0.012)	−0.589 (0.002)	−0.629 (0.000)	0.042 (NS)	−0.321(NS)	−0.297 (NS)	−0.334 (NS)	0.150 (NS)	−0.263 (NS)
lncRNA-p19461	−0.536 (0.006)	−0.438 (0.018)	−0.321 (NS)	−0.115 (NS)	−0.428 (0.029)	−0.422 (0.036)	−0.241 (NS)	0.173 (NS)	0.073 (NS)

NS, not significant.

**Table 3:** Clinical characteristics of subjects included in longitudinal studies.

	Baseline	After weight lost	Variation, %	*P*
BMI (kg/m^2^)	32.63 ± 4.28	29.86 ± 4.38	−8.49	0.001
Waist circumference (cm)	103.67 ± 9.95	100.63 ± 9.65	−2.93	0.000
Waist-to-hip ratio	0.93 ± 0.05	0.91 ± 0.05	−2.15	0.013
Fasting glucose (mmol/L)	5.35 ± 0.63	5.14 ± 0.40	−3.93	0.408
Fasting insulin (μU/mL)	15.48 ± 11.10	9.79 ± 6.16	−36.76	0.015
HOMA-β (%)	168.59 ± 100.71	116.97 ± 49.34	−30.62	0.066
HOMA-IR	3.82 ± 3.04	2.30 ± 1.68	−39.79	0.019
lncRNA-p5549	0.15 ± 0.08	0.19 ± 0.10	26.67	0.355
lncRNA-p21015	0.49 ± 0.34	0.86 ± 0.69	75.51	0.169
lncRNA-p19461	0.40 ± 0.20	1.68 ± 0.63	320.00	0.001
